# Enzymatic Treatment of Sprouted Wheat and Buckwheat Modulates In Vitro Starch Digestibility and Technological Quality of Wheat Bread

**DOI:** 10.3390/foods15213976

**Published:** 2026-11-05

**Authors:** Darigash Shaimerdenova, Zhanar Chakanova, Sandugash Anuarbekova, Damira Iskakova, Gaini Sarbassova, Maigul Kizatova, Akzharkyn Nurmanova, Kuanysh Kurtibay, Baltash Tarabayev

**Affiliations:** 1 LLP “Research and Production Enterprise “Innovator”, Astana 010000, Kazakhstan; darigash.sh@gmail.com (D.S.);; 2 Department of Pharmaceutical Technology, S.D. Asfendiyarov Kazakh National Medical University, Almaty 050000, Kazakhstan; 3 Technology of Food and Processing Industries, Saken Seifullin Kazakh Agrotechnical University, Astana 010000, Kazakhstan

**Keywords:** resistant starch, slowly digestible starch, rapidly digestible starch, α-amylase, glucoamylase, cereal bioprocessing

## Abstract

This study evaluated whether sprouting followed by amylolytic enzyme treatment can modify starch digestibility in wheat bread while assessing associated changes in technological quality. Wheat and common buckwheat were germinated for 12–48 h; 24 h was selected for subsequent treatment with α-amylase and glucoamylase. Based on preliminary screening, wheat was treated at 35 °C for 12 h and buckwheat at 40 °C for 12 h. Sprouted or enzyme-treated sprouted ingredients were incorporated into wheat bread at 10, 15, and 20% of flour mass. Rapidly digestible starch (RDS), slowly digestible starch (SDS), resistant starch (RS), loaf volume, porosity, and titratable acidity were evaluated. In bread containing enzyme-treated sprouted wheat, increasing inclusion from 10 to 20% decreased RDS from 76.58 to 73.42% and increased SDS from 14.05 to 17.00%; a second independent series showed the same pattern. Enzyme-treated sprouted buckwheat produced higher RS values, reaching 11.61% at 10% inclusion. Wheat-based enzyme-treated formulations also retained comparatively favorable loaf volumes, whereas higher buckwheat inclusion reduced volume. These results indicate crop-specific effects of enzymatic pretreatment and support further evaluation of enzyme-treated sprouted cereals as ingredients for modulating in vitro starch digestibility in bread.

## 1. Introduction

Wheat bread is a major source of dietary starch, and its nutritional behavior depends not only on total starch content but also on the rate at which starch is hydrolyzed during digestion. The Englyst classification separates starch into rapidly digestible starch (RDS), slowly digestible starch (SDS), and resistant starch (RS), providing a practical framework for comparing starch-rich foods with different digestion profiles [1,2,3]. Starch digestibility is also influenced by processing and by the physical and compositional characteristics of the food matrix [4]. RS is widely studied as a fermentable dietary fiber and as a functional component of bread formulations [5,6]; however, in vitro RDS, SDS, and RS values should not be interpreted as direct evidence of postprandial glycemic effects.

Sprouting is one approach for modifying cereal starch before baking. Activation of endogenous hydrolytic enzymes during germination changes starch content, sugar formation, and the organization of starch granules. Recent work has shown that germination time can alter RDS, SDS, and RS in cereal matrices and can also affect dough behavior and bread quality [7,8,9,10]. However, the direction and magnitude of these changes depend on the cereal species, sprouting intensity, inclusion level, and subsequent processing steps [10].

Wheat (*Triticum aestivum* L.) and common buckwheat (*Fagopyrum esculentum Moench*) differ in starch structure and composition. Comparative studies have linked differences in starch digestibility between wheat and buckwheat to molecular characteristics, including amylopectin chain distribution, while recent reviews and multi-variety studies emphasize substantial genotype- and matrix-dependent variability in buckwheat starch properties [11,12,13,14,15]. These differences make the two crops useful models for evaluating whether the same biotechnological treatment produces comparable effects in bread.

A second level of modification can be achieved by treating sprouted grain with amylolytic enzymes. α-Amylase and glucoamylase alter starch molecular structure and hydrolysis behavior, and their effects depend strongly on enzyme system, treatment intensity, and substrate characteristics [16,17,18]. In bread systems, amylase-mediated modification can influence both starch digestibility and technological properties, indicating that nutritional and processing outcomes should be evaluated together [19].

The present study evaluated sprouted and enzyme-treated sprouted wheat and buckwheat as partial replacements for wheat flour. The objectives were (i) to select practical germination and enzyme-treatment conditions from preliminary screening, (ii) to compare the effects of sprouted and enzyme-treated sprouted cereal ingredients on RDS, SDS, and RS in wheat bread, and (iii) to determine whether changes in starch digestibility were accompanied by major changes in loaf volume, crumb porosity, and titratable acidity.

## 2. Materials and Methods

### 2.1. Materials

Soft wheat (*Triticum aestivum* L.) and common buckwheat (*Fagopyrum esculentum Moench*) were used as cereal raw materials. Wheat flour (‘Ramazan’, premium grade; Aktobe, Kazakhstan), instant dry baker’s yeast (Pakmaya, Pak Gıda Üretim ve Pazarlama A.Ş., Istambul, Türkiye), food-grade salt, sucrose, and potable water were used for bread preparation.

For enzymatic treatment, α-amylase GRM638 and glucoamylase GRM7062 (HiMedia Laboratories Pvt. Ltd., Thane, India) were used. For in vitro starch digestion, pancreatic α-amylase Type VI-B (A3176, Sigma-Aldrich, Louis, MO, USA) and glucoamylase from *Aspergillus niger* (GRM7062, HiMedia Laboratories Pvt. Ltd., Mumbai, India) were used. D-(+)-glucose (D9434, Sigma-Aldrich, USA) was used for calibration of the DNS assay.

### 2.2. Experimental Design and Selection of Processing Conditions

The study was conducted in two stages. In the preliminary stage, wheat and buckwheat were germinated for 12, 24, 36, and 48 h to identify a practical germination time for subsequent enzymatic treatment (Tables S1 and S2). Based on the balance between starch loss and the development of measurable changes in the carbohydrate profile, 24 h germination was selected.

The 24 h sprouted grain was subsequently treated with α-amylase and glucoamylase under two temperature schedules: 35 °C for 12, 24, or 36 h and 40 °C for 6, 12, or 18 h. The conditions selected for breadmaking were 35 °C for 12 h for wheat and 40 °C for 12 h for buckwheat. These conditions produced clear enzymatic modification while avoiding the more extensive starch degradation observed at longer treatment times.

For breadmaking, sprouted wheat, sprouted buckwheat, enzyme-treated sprouted wheat, and enzyme-treated sprouted buckwheat were incorporated at nominal levels equivalent to 10, 15, or 20% of the flour mass. Two independent experimental series of enzyme-treated ingredients were used to assess the reproducibility of the observed direction of response.

### 2.3. Grain Decontamination and Germination

Before germination, cereal grains were surface-decontaminated. Grain was treated with 70% ethanol for 2–5 min with continuous mixing, followed by 4% sodium hypochlorite (300–400 mL per 100 g grain) at 20 ± 1 °C and 200 rpm for 50 min. The grain was washed at least five times with sterile distilled water, treated with 0.01 N HCl for 10 min to neutralize residual hypochlorite, and washed at least three additional times. Grain was then soaked in water at a grain-to-water ratio of 1:2 for 24 h at 20 °C, drained, distributed on moistened cotton cloth, and germinated at 25 ± 1 °C and approximately 85% relative humidity. Samples were collected after 12, 24, 36, and 48 h; 24 h material was used for enzymatic treatment. Surface decontamination followed the general rationale used to reduce seed-borne microbial contamination during sprouting [20].

### 2.4. Enzymatic Treatment of Sprouted Grain

A working α-amylase solution was prepared by dissolving 1.0 g of GRM638 in acetate buffer (pH 5.2) to a final volume of 50 mL. A working glucoamylase solution was prepared from 0.1 g of GRM7062 and diluted to 50 mL with the same buffer. Fresh solutions were added at 1 mL α-amylase solution and 1 mL glucoamylase solution per 100 mL aqueous phase. α-Amylase and glucoamylase solutions were prepared using an acetate buffer at pH 5.2. The mildly acidic medium was selected to provide suitable conditions for the combined action of the amylolytic enzymes. According to Guo et al. [21] α-amylase and glucoamylase have different optimal pH values; however, a mildly acidic environment with a pH of approximately 5.0 can be used to support their simultaneous action during starch hydrolysis. Enzymatic treatment of the sprouted grain was carried out in sterile distilled water supplemented with the prepared enzyme solutions. The pH of the reaction mixture was measured using a pH meter and was 5.4 at the end of the treatment. Enzymatic treatment was carried out at a sprouted grain-to-liquid phase ratio of 1:3 (*w*/*v*). For each 100 g of sprouted grain, 300 mL of sterile distilled water was added.

Based on preliminary screening, wheat treated at 35 °C for 12 h and buckwheat treated at 40 °C for 12 h were selected for breadmaking. After treatment, the material was homogenized and used directly in the dough in its prepared liquid form. The commercial enzyme preparations were dosed using the mass-based preparation, dilution, and addition procedure described above; standardized activity units were not available for reporting enzyme dose. According to the manufacturer’s technical specifications, the activity of malt-derived α-amylase (GRM638) is not less than 1:2000, as determined according to the Indian Pharmacopoeia (IP) method, whereas the activity of glucoamylase from Aspergillus niger (RM7062, HiMedia Laboratories Pvt. Ltd., Mumbai, India) is not less than 30 U/mg of dry matter at pH 4.8 and 60 °C. These values correspond to the activity-assay conditions specified by the manufacturer.

### 2.5. Bread Preparation

Bread was prepared according to the methodological principles of ICC Standard No. 131 and AACC International Method 10-10.03 [22,23], with modifications to the formulation and processing parameters required by the experimental design.

Sprouted wheat and buckwheat grains were used without prior drying or milling, in the form of a liquid homogenized suspension, following the technological approach described by Akiki et al. [24]. Wheat flour was replaced with wet sprouted grain at levels of 10, 15, and 20% of the initial flour mass (300 g). Accordingly, 270, 255, and 240 g of wheat flour were combined with 30, 45, and 60 g of wet sprouted grain, respectively. The control sample KX01 was prepared using the same base formulation, without replacement of wheat flour by the investigated cereal ingredients (0% replacement). The suspension was prepared at a grain-to-sterile distilled water ratio of 1:3 (*w*/*v*), and 120, 180, and 240 g of suspension were incorporated into the dough, respectively. Grain suspensions were used either without enzymatic treatment or after treatment with α-amylase and glucoamylase as described in Section 2.4. The formulation compositions are presented in Table 1.

The dough was mixed manually with 6 g of dry baker’s yeast, 5.4 g of salt, and 6 g of sucrose. For the preparation of the KX01 control sample, 200 mL of water was used. In the experimental formulations with 10, 15, and 20% replacement of wheat flour, the amounts of water introduced with the grain suspension were 90, 135, and 180 mL, respectively, while the corresponding amounts of additional water added during dough preparation were 110, 65, and 20 mL. Thus, the total amount of added water was maintained at 200 mL in all formulations, including the water used to prepare the grain suspension. Fermentation was carried out at 30 °C for 90 min, followed by proofing at 37 °C and 80% relative humidity for 40–50 min. Baking was performed at 200 °C for 30 min. After baking, the bread was cooled at room temperature. Each formulation was prepared in three independent baking runs. The initial amount of water was calculated taking into account the actual moisture content of the wheat flour, following the methodological approach described in GOST 27669-88 [25]. For the experimental formulations, the amount of water was further adjusted during mixing to achieve the required technological consistency of the dough.

### 2.6. Determination of Total Strach and Starch Digestibility Fractions

Total starch (TS) content was determined using the Ewers polarimetric method in accordance with ISO 6493:2000 [26]. Samples of bread crumb, raw grain, and sprouted grain were dried at 105 °C, whereas enzyme-treated samples were dried in a vacuum oven at 40 °C to constant mass. The dried samples were ground to pass through a 0.5 mm sieve.

A 2.5 g portion of each sample was treated with 0.31 mol/L HCl in a boiling water bath for 15 min, clarified with Carrez I and Carrez II solutions, and filtered. Optical rotation was measured using an SM-3 polarimeter at a wavelength of 589.3 nm with a 200 mm observation tube. To account for the contribution of soluble sugars and dextrins, the optical rotation of an extract obtained by treating a 5 g sample with 40% ethanol was determined separately. Starch content was calculated using Equation (1), applying specific optical rotation values of +182.7° for wheat starch and +184.0° for buckwheat starch and the corresponding mixtures. Analyses were performed in triplicate, and the results were expressed on a dry-matter basis.

(1) $$TS=\frac{2000}{{\left[\alpha \right]}_{D}^{20}}\times \left(\frac{2.5\alpha_{1}}{m_{1}}-\frac{5\alpha_{2}}{m_{2}}\right)$$

RDS, SDS, and RS were determined using an in vitro approach based on Englyst, Goux [3]. The mass of each bread sample was adjusted to contain 20 mg total starch and suspended in 15 mL of 0.1 M acetate buffer (pH 5.2) preheated to 37 °C. A 0.5 mL aliquot was removed before enzyme addition and transferred immediately into 10 mL absolute ethanol to determine free glucose (FG).

Hydrolysis was performed using pancreatic α-amylase Type VI-B and glucoamylase from *Aspergillus niger*. Samples were incubated at 37 ± 0.5 °C with shaking at 160 rpm. Aliquots were collected after 20 and 120 min, the reaction was stopped, and samples were centrifuged at 4000 rpm for 15 min at 20 °C. Glucose in the supernatant was quantified by the 3,5-dinitrosalicylic acid (DNS) method at 540 nm using a D-(+)-glucose calibration curve (0–1.0 mg/mL; R^2^ ≥ 0.995).

$$RDS=\frac{\left(G_{20}-FG\right)\times 0.9\;}{TS}\times 100$$

$$SDS=\frac{\left(G_{120}-G_{20}\right)\times 0.9\;}{TS}\times 100$$

RS=100−RDS−SDS

where *G*_20_ and *G*_120_ are glucose released after 20 and 120 min, respectively, FG is free glucose, TS is total starch in the test portion, and 0.9 converts glucose to starch equivalents. For each independent baking run, starch-digestibility measurements were performed in triplicate analytical determinations, and the analytical values were averaged to obtain one value per baking run. A reagent blank was included in each analytical series.

### 2.7. Technological Quality of Bread

Bread mass was determined gravimetrically. Loaf volume was measured by the rapeseed displacement method according to AACC International Method 10-05.01 [27]. Crumb porosity was determined according to GOST 5669-96 [28], and titratable acidity was determined according to GOST 5670-96 [29]. These parameters were used to assess the technological quality of the bread.

### 2.8. Statistical Analysis

Each bread formulation was produced in three independent baking runs. Results are presented as mean ± standard deviation (SD; *n* = 3), where n represents independent baking replicates. Technical analytical replicates were averaged to obtain one value for each independent baking run. For the parameters reported in Table 2 and Table 3, statistical analyses were performed in accordance with the structure of the experimental design. The effects of cereal type, processing method, and ingredient inclusion level on loaf volume, crumb porosity, and titratable acidity were evaluated using three-way analysis of variance (ANOVA), including interactions among the factors. To compare individual formulations, one-way ANOVA was performed with formulation treated as a factor with 19 levels (18 experimental formulations and the KX01 control), followed by Tukey’s honestly significant difference (HSD) test for multiple comparisons. Statistical significance was set at *p* ≤ 0.05. To further assess the comparability of the two independent experimental series (S1 and S2), a two-way ANOVA was performed. The analysis was conducted separately for wheat and buckwheat, with experimental series (S1 and S2) and the inclusion level of enzyme-treated sprouted material (10, 15, and 20%) considered as two factors. The main effects of both factors and their interaction were evaluated for RDS, SDS, and RS contents, as well as loaf volume, crumb porosity, and titratable acidity. The results are presented in Supplementary Table S4. Statistical analyses were performed using Statistica 13 software (StatSoft Inc., Tulsa, OK, USA).

## 3. Results

### 3.1. Preliminary Selection of Germination and Enzyme-Treatment Conditions

Germination progressively decreased total starch and increased reducing sugars in both cereals. In wheat, total starch decreased from 63.1 ± 1.0% in ungerminated grain to 58.9 ± 1.3% after 24 h and 53.8 ± 1.5% after 48 h. In buckwheat, the corresponding values were 59.8 ± 1.1, 53.0 ± 1.4, and 45.7 ± 1.3%. RDS increased during germination, while SDS and RS decreased. After 24 h, wheat contained 36.8 ± 1.5% RDS, 47.0 ± 1.9% SDS, and 16.1 ± 0.8% RS; buckwheat contained 42.5 ± 1.7% RDS, 42.6 ± 1.7% SDS, and 15.1 ± 0.7% RS. The 24 h time point was therefore retained as an intermediate condition that produced a clear carbohydrate shift without the more extensive starch depletion observed after 36–48 h. Thus, germination was accompanied by a reduction in total starch content and changes in the relative distribution of starch fractions in both cereal species. The magnitude of these changes depended on the duration of germination and the type of grain. Detailed results on the dynamics of carbohydrate composition in wheat and buckwheat are presented in Supplementary Table S1.

Longer enzymatic treatment further reduced total starch and shifted the starch profile toward RDS. At the selected wheat condition (35 °C, 12 h), total starch was 42.7 ± 1.1%, with RDS, SDS, and RS representing 52.5 ± 2.5, 35.8 ± 2.1, and 11.7 ± 1.0% of total starch, respectively. At the selected buckwheat condition (40 °C, 12 h), total starch was 29.6 ± 1.1%, with RDS, SDS, and RS of 73.6 ± 3.8, 20.9 ± 1.9, and 5.4 ± 0.7%, respectively. More prolonged treatment produced stronger hydrolysis and was not used for the bread formulations. Enzymatic treatment of sprouted grain resulted in further changes in carbohydrate composition, the magnitude of which depended on the treatment temperature and duration. These results were used to justify the selection of processing conditions for the preparation of the experimental bread samples. Detailed data on changes in carbohydrate composition under different enzymatic treatment conditions are presented in Supplementary Table S2.

### 3.2. Starch Digestibility of Bread

**Figure 1:**
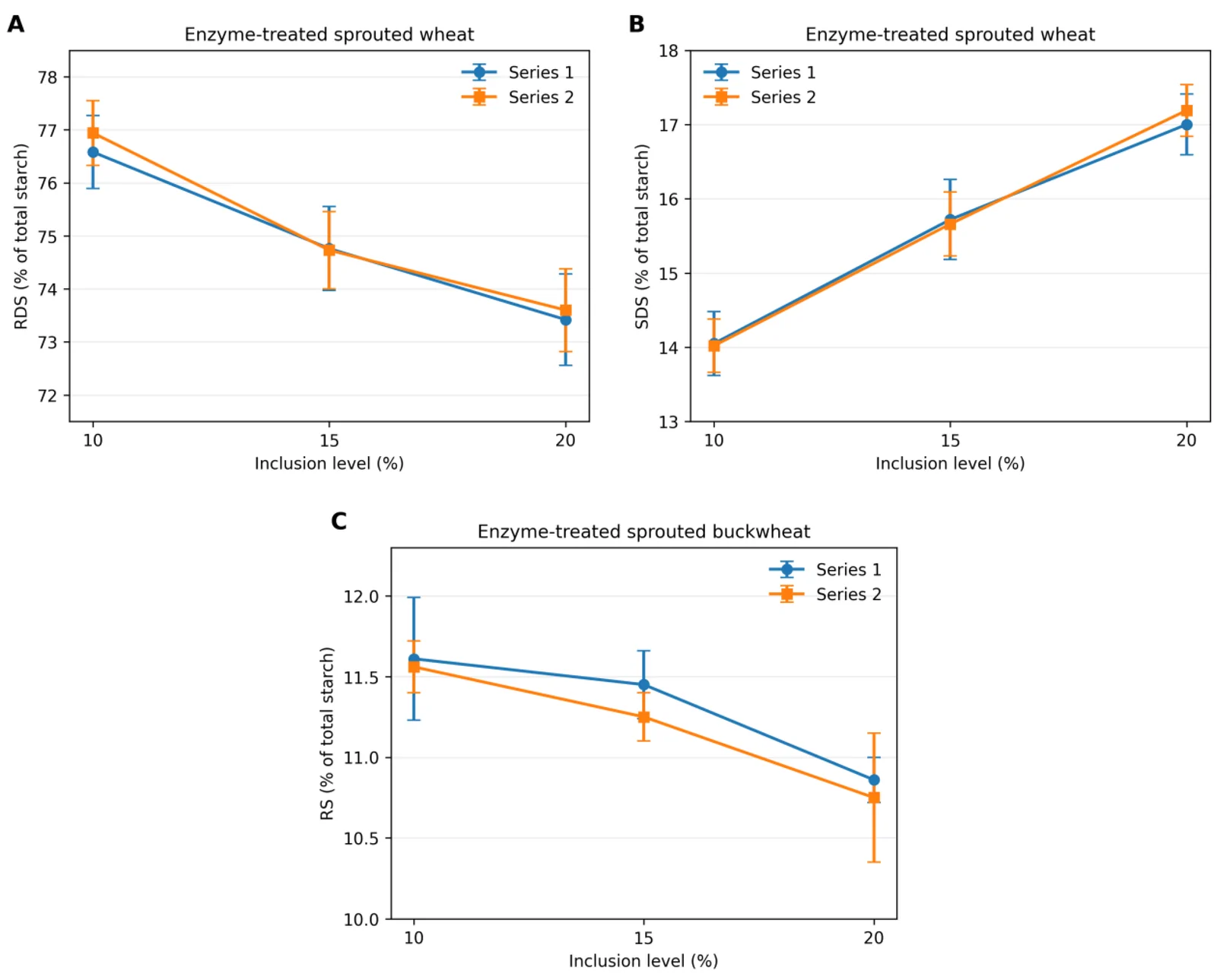
Reproducibility of the principal changes in starch digestibility in breads containing enzyme-treated sprouted wheat and buckwheat. (**A**) Rapidly digestible starch (RDS) in breads containing 10, 15, and 20% enzyme-treated sprouted wheat; (**B**) slowly digestible starch (SDS) in the corresponding wheat formulations; and (**C**) resistant starch (RS) in breads containing 10, 15, and 20% enzyme-treated sprouted buckwheat. S1 and S2 denote independent experimental series 1 and 2. Values are presented as mean ± SD (*n* = 3 independent baking runs). Lines illustrate the direction of response across inclusion levels and should not be interpreted as fitted dose–response models.

Formulation significantly affected RDS, SDS, and RS (*p* < 0.001 for each response; Table 2). The clearest treatment-dependent response occurred in bread containing enzyme-treated sprouted wheat (Figure 1A,B; Table 2). In series 1, increasing inclusion from 10 to 20% decreased RDS from 76.58 ± 0.69 to 73.42 ± 0.86% of total starch while increasing SDS from 14.05 ± 0.43 to 17.00 ± 0.41%. Series 2 reproduced the same direction, with RDS decreasing from 76.94 ± 0.61 to 73.60 ± 0.78% and SDS increasing from 14.02 ± 0.36 to 17.19 ± 0.35%. RS remained comparatively stable in the wheat formulations.

Sprouted wheat without enzyme treatment showed a much smaller dose-related change: RDS remained between 78.54 and 78.85%, SDS between 11.87 and 12.26%, and RS between 9.20 and 9.37%. Thus, the repeated shift toward SDS was associated specifically with the enzyme-treated sprouted wheat rather than with sprouting alone.

Buckwheat showed a different pattern (Figure 1C; Table 2). Sprouted buckwheat breads contained 8.97–9.35% RS, whereas enzyme-treated sprouted buckwheat reached 11.61 ± 0.38% RS at 10% inclusion in series 1 and 11.56 ± 0.16% in series 2. RS then declined modestly as inclusion increased to 20%. The two independent series were highly consistent in the direction and approximate magnitude of this response, indicating that enzyme-treated buckwheat preferentially affected RS rather than SDS.

The reproducibility of the principal starch-digestibility responses across the two independent experimental series is illustrated in Figure 1. In enzyme-treated sprouted wheat, increasing the inclusion level produced a consistent decrease in RDS and increase in SDS in both series, whereas enzyme-treated sprouted buckwheat showed the highest RS content at the 10% inclusion level, followed by a modest decline at higher inclusion levels. Two-way ANOVA confirmed a statistically significant effect of ingredient inclusion level on RDS and SDS in wheat bread and on RS in buckwheat bread (*p* < 0.001). No significant differences were detected between the two independent experimental series (*p* = 0.353–0.871), and no significant interaction between experimental series and inclusion level was observed (*p* = 0.858–0.904). These findings indicate that the observed response patterns were consistent across both experimental series (Figure 1).

### 3.3. Technological Quality of Bread

Formulation had a statistically significant effect on loaf volume, crumb porosity, and titratable acidity (*p* < 0.001 for each parameter; Table 3). The control sample KX01 had a loaf volume of 314 ± 7.9 cm^3^, a crumb porosity of 78 ± 1.7%, and a titratable acidity of 1.80 ± 0.26 °H. Technological quality depended on both cereal species and treatment. For sprouted wheat, loaf volume ranged from 290 to 315 cm^3^. Enzyme-treated sprouted wheat retained or improved volume in several formulations: BL27 (20%, series 1) reached 340 ± 7.9 cm^3^, while FR31 and NJ34 in series 2 both reached 347 cm^3^. Porosity in the wheat formulations ranged from 68 to 78%.

Buckwheat formulations showed a greater tendency toward reduced loaf volume at higher inclusion. Sprouted buckwheat decreased from 307 ± 5.3 cm^3^ at 10% to 280 ± 10.6 cm^3^ at 20%. In enzyme-treated buckwheat, the highest observed volume was 323 ± 5.3 cm^3^ at 10% inclusion (series 1), while 20% inclusion yielded 286 ± 5.3 cm^3^ in series 1 and 283 ± 5.3 cm^3^ in series 2. These findings indicate that the starch-digestibility response of enzyme-treated buckwheat was accompanied by a stronger technological trade-off than the response observed with enzyme-treated wheat. To assess the combined effects of technological factors on bread quality, a three-way analysis of variance was performed. A statistically significant interaction among cereal type, processing method, and ingredient inclusion level was observed for loaf volume (F(2,24) = 15.68, *p* < 0.001), crumb porosity (F(2,24) = 38.38, *p* < 0.001), and titratable acidity (F(2,24) = 6.24, *p* = 0.007). These findings indicate that the effect of ingredient inclusion level on the technological properties of bread depends on both the cereal type and the processing method. Detailed results of the factorial analysis are provided in Supplementary Table S3.

## 4. Discussion

The results show that sprouting and subsequent enzymatic treatment should not be treated as interchangeable processing steps. Germination alone activated starch hydrolysis and progressively shifted the grain profile toward higher RDS and lower SDS/RS, consistent with the activation of endogenous enzymes and structural disruption of starch during sprouting [7,8]. Studies of sprouted wheat flour and bread likewise show that the extent of germination and the associated increase in amylolytic activity can substantially alter dough rheology and final bread quality [9,10]. For that reason, an intermediate germination period of 24 h was selected before exogenous enzyme treatment rather than extending germination to the point of greater starch depletion.

The most reproducible bread-level response was observed with enzyme-treated sprouted wheat. Across both independent experimental series, increasing inclusion from 10 to 20% was accompanied by an approximately three percentage-point decrease in RDS and a similar increase in SDS (Figure 1A,B). Sprouted wheat without enzyme treatment did not show this pattern. The results are consistent with previous reports indicating that controlled starch modification can influence starch digestibility [17,30]. Similar changes in starch digestibility in wheat bread following treatment with amylolytic enzymes have also been reported [19]. The present data do not establish the molecular mechanism, but the repeated direction of response across two series strengthens the interpretation that enzymatic pretreatment, rather than sprouting alone, was responsible for the shift toward SDS.

Buckwheat behaved differently. Enzyme-treated sprouted buckwheat produced higher RS than the corresponding sprouted-only formulations, with the highest RS observed at 10% inclusion (Figure 1C). Increasing the amount of enzyme-treated buckwheat did not increase RS further. This non-linear response is plausible given the distinct molecular characteristics of buckwheat starch, the documented variability among buckwheat genotypes, and the contribution of protein and lipid interactions to starch accessibility [11,15]. The contrast between wheat and buckwheat therefore supports a crop-specific response to the same general processing concept rather than a universal effect of enzyme treatment.

The technological characteristics of the bread complement the findings on starch digestibility. Samples containing enzyme-treated sprouted wheat, particularly those from the second experimental series, were characterized by comparatively high loaf volumes and an increased proportion of SDS. In the buckwheat formulations, increasing the inclusion level was associated with a reduction in loaf volume despite the higher RS content compared with samples containing sprouted buckwheat without enzymatic treatment. Comparison with the KX01 control provided additional context for evaluating differences in the technological characteristics of the breads. However, the observed changes should not be interpreted unequivocally as an improvement or deterioration in quality relative to breads containing the corresponding unmodified cereal ingredients. These findings are consistent with previous reports showing that cereal-matrix composition and structure influence starch accessibility and digestibility [31,32,33], as well as with evidence that physicochemical transformations during baking play an important role in determining loaf volume and bread structure [34]. The differences observed between the independent experimental series indicate a certain degree of variability in the technological characteristics of the bread, particularly loaf volume, porosity, and titratable acidity. At the same time, the absence of a statistically significant effect of experimental series on RDS, SDS, and RS contents suggests that the investigated starch-digestibility parameters were relatively stable across series. These findings highlight the importance of accounting for inter-series variability when evaluating the technological quality of bread, without automatically extending such variability to the carbohydrate-composition characteristics.

The study has several limitations that should be considered when interpreting the findings. First, sensory evaluation was not performed; therefore, the practical acceptability of the breads, particularly at higher inclusion levels of the modified cereal ingredients, could not be assessed. Second, although the KX01 control was included, the modified ingredients were not fully standardized on a dry-matter basis, which limits the ability to unequivocally distinguish the effects of enzymatic treatment from those associated with differences in formulation composition. Third, farinographic analysis was not performed; consequently, water absorption and dough rheological properties were not quantitatively characterized. This limits a more detailed interpretation of the relationship between ingredient incorporation, dough behavior, and the observed technological characteristics of the breads. Therefore, the results should be interpreted within the specific experimental conditions used in this study. Despite these limitations, the observed trends were supported by two complementary findings: enzyme-treated sprouted wheat reproducibly shifted the bread starch profile toward SDS, whereas enzyme-treated sprouted buckwheat preferentially increased RS at lower inclusion levels. The species-specific response, together with the associated differences in loaf quality, provides a practical basis for further optimization using fully standardized enzyme activity, dry-matter-corrected formulations, and independent production replicates.

## 5. Conclusions

Enzyme-treated sprouted wheat consistently reduced RDS and increased SDS as its inclusion level increased, and this pattern was reproduced in two independent experimental series. Enzyme-treated sprouted buckwheat produced a different response, with RS values of approximately 11.5–11.6% at 10% inclusion and a modest decline at higher levels. Wheat formulations generally retained better loaf volume than buckwheat formulations. These findings suggest that the observed changes in bread starch digestibility may depend on the cereal ingredient used and should be evaluated together with technological quality. The results support further development of enzyme-treated sprouted cereals as ingredients for wheat bread with a deliberately modified in vitro starch-digestion profile.

## Figures and Tables

**Table 1:** Experimental bread formulations.

Code	Formulation
KX01	Control wheat bread, 0
DF11	10% sprouted wheat
RT14	15% sprouted wheat
HG17	20% sprouted wheat
VM21	10% enzyme-treated sprouted wheat, series 1
ZS24	15% enzyme-treated sprouted wheat, series 1
BL27	20% enzyme-treated sprouted wheat, series 1
FR31	10% enzyme-treated sprouted wheat, series 2
NJ34	15% enzyme-treated sprouted wheat, series 2
YC37	20% enzyme-treated sprouted wheat, series 2
GK41	10% sprouted buckwheat
HM44	15% sprouted buckwheat
QR47	20% sprouted buckwheat
ST51	10% enzyme-treated sprouted buckwheat, series 1
VW54	15% enzyme-treated sprouted buckwheat, series 1
ZX57	20% enzyme-treated sprouted buckwheat, series 1
AB61	10% enzyme-treated sprouted buckwheat, series 2
CD64	15% enzyme-treated sprouted buckwheat, series 2
EF67	20% enzyme-treated sprouted buckwheat, series 2

**Table 2:** Contents of rapidly digestible starch (RDS), slowly digestible starch (SDS), and resistant starch (RS) in the control and experimental bread samples.

Code	RDS (% TS)	SDS (% TS)	RS (% TS)
KX01	79.19 ± 0.91 ^a^	11.70 ± 0.37 ^f^	9.11 ± 0.14 ^d^
DF11	78.62 ± 0.54 ^ab^	12.01 ± 0.29 ^def^	9.37 ± 0.25 ^d^
RT14	78.85 ± 0.66 ^ab^	11.87 ± 0.33 ^ef^	9.28 ± 0.23 ^d^
HG17	78.54 ± 0.91 ^ab^	12.26 ± 0.49 ^def^	9.20 ± 0.17 ^d^
VM21	76.58 ± 0.69 ^bcde^	14.05 ± 0.43 ^c^	9.37 ± 0.20 ^d^
ZS24	74.76 ± 0.79 ^ef^	15.72 ± 0.54 ^b^	9.52 ± 0.26 ^d^
BL27	73.42 ± 0.86 ^f^	17.00 ± 0.41 ^a^	9.58 ± 0.18 ^d^
FR31	76.94 ± 0.61 ^abcde^	14.02 ± 0.36 ^c^	9.04 ± 0.27 ^d^
NJ34	74.73 ± 0.73 ^ef^	15.66 ± 0.43 ^b^	9.61 ± 0.24 ^d^
YC37	73.60 ± 0.78 ^f^	17.19 ± 0.35 ^a^	9.21 ± 0.19 ^d^
GK41	78.35 ± 0.57 ^ab^	12.59 ± 0.26 ^def^	9.06 ± 0.22 ^d^
HM44	78.19 ± 0.68 ^abc^	12.84 ± 0.36 ^cdef^	8.97 ± 0.18 ^d^
QR47	77.51 ± 0.93 ^abcd^	13.14 ± 0.32 ^cd^	9.35 ± 0.25 ^d^
ST51	75.21 ± 0.72 ^ef^	13.18 ± 0.38 ^cd^	11.61 ± 0.38 ^a^
VW54	75.65 ± 0.79 ^def^	12.90 ± 0.45 ^cdef^	11.45 ± 0.21 ^abc^
ZX57	76.69 ± 0.69 ^bcde^	12.45 ± 0.53 ^def^	10.86 ± 0.14 ^bc^
AB61	75.39 ± 0.58 ^def^	13.05 ± 0.34 ^cde^	11.56 ± 0.16 ^ab^
CD64	76.05 ± 0.83 ^cde^	12.70 ± 0.41 ^def^	11.25 ± 0.15 ^abc^
EF67	76.89 ± 0.75 ^bcde^	12.36 ± 0.49 ^def^	10.75 ± 0.40 ^c^

Values are presented as mean ± SD (*n* = 3 independent baking runs). Within each column, means sharing at least one superscript letter are not significantly different; means with no letter in common differ according to Tukey’s HSD test (*p* ≤ 0.05). One-way analysis of variance (ANOVA): RDS, F(18,38) = 16.99, *p* < 0.001; SDS, F(18,38) = 51.83, *p* < 0.001; RS, F(18,38) = 52.35, *p* < 0.001. S1 and S2 denote independent experimental series 1 and 2.

**Table 3:** Technological characteristics of the control and experimental bread samples.

Code	Loaf Volume, cm^3^	Porosity, %	Titratable Acidity, °H
KX01	314 ± 7.9 ^cde^	78 ± 1.7 ^a^	1.80 ± 0.26 ^cd^
DF11	294 ± 5.3 ^efgh^	69 ± 1.0 ^cde^	2.00 ± 0.26 ^bcd^
RT14	315 ± 5.3 ^cde^	70 ± 1.7 ^cd^	2.20 ± 0.26 ^abcd^
HG17	290 ± 7.9 ^fgh^	72 ± 1.0 ^bc^	2.40 ± 0.26 ^abc^
VM21	297 ± 10.6 ^efgh^	78 ± 1.7 ^a^	1.60 ± 0.20 ^d^
ZS24	313 ± 5.3 ^def^	68 ± 1.0 ^cde^	1.60 ± 0.26 ^d^
BL27	340 ± 7.9 ^ab^	71 ± 1.7 ^bc^	1.80 ± 0.26 ^cd^
FR31	347 ± 5.3 ^a^	71 ± 1.7 ^bc^	2.00 ± 0.26 ^bcd^
NJ34	347 ± 10.6 ^a^	70 ± 1.7 ^cd^	2.20 ± 0.26 ^abcd^
YC37	337 ± 10.6 ^abc^	70 ± 1.7 ^cd^	2.20 ± 0.26 ^abcd^
GK41	307 ± 5.3 ^defg^	75 ± 1.0 ^ab^	2.80 ± 0.26 ^a^
HM44	284 ± 7.9 ^gh^	69 ± 1.7 ^cde^	2.00 ± 0.26 ^bcd^
QR47	280 ± 10.6 ^h^	66 ± 1.7 ^de^	2.60 ± 0.26 ^ab^
ST51	323 ± 5.3 ^bcd^	70 ± 1.0 ^cd^	2.00 ± 0.20 ^bcd^
VW54	297 ± 7.9 ^efgh^	71 ± 1.0 ^bc^	2.40 ± 0.20 ^abc^
ZX57	286 ± 5.3 ^gh^	68 ± 1.7 ^cde^	2.60 ± 0.26 ^ab^
AB61	287 ± 5.3 ^gh^	68 ± 1.7 ^cde^	2.40 ± 0.26 ^abc^
CD64	288 ± 7.9 ^gh^	72 ± 1.7 ^bc^	2.80 ± 0.26 ^a^
EF67	283 ± 5.3 ^h^	65 ± 1.0 ^e^	2.80 ± 0.26 ^a^

Values are presented as mean ± SD (*n* = 3 independent baking runs). Within each column, means sharing at least one superscript letter are not significantly different; means with no letter in common differ according to Tukey’s HSD test (*p* ≤ 0.05). One-way analysis of variance (ANOVA): loaf volume, F(18,38) = 27.28, *p* < 0.001; porosity, F(18,38) = 15.66, *p* < 0.001; titratable acidity, F(18,38) = 6.92, *p* < 0.001. S1 and S2 denote independent experimental series 1 and 2. Two-way ANOVA revealed a statistically significant effect of experimental series on loaf volume for both wheat and buckwheat breads (*p* < 0.001), on titratable acidity (*p* = 0.002 and *p* = 0.014, respectively), and on porosity of the wheat bread (*p* = 0.023). For buckwheat bread porosity, the effect of experimental series did not reach statistical significance (*p* = 0.069). In addition, a significant interaction between experimental series and ingredient inclusion level was observed for loaf volume in both cereal groups and for porosity in the wheat formulations (Table S4).

## Data Availability

The original contributions presented in this study are included in the article/Supplementary Material. Further inquiries can be directed to the corresponding authors.

## Supplementary Materials

The following supporting information can be downloaded at: https://www.mdpi.com/article/10.3390/foods15213976/s1, Table S1: Changes in the carbohydrate composition of wheat and buckwheat during germination (0–48 h); Table S2: Changes in the carbohydrate composition of wheat and buckwheat germinated for 24 h during enzymatic treatment under different temperature–time conditions; Table S3: Results of three-way analysis of variance for the effects of cereal type, processing method, and ingredient inclusion level on the technological quality characteristics of bread; Table S4: Results of two-way analysis of variance (ANOVA): effects of experimental series and ingredient inclusion level.
