# Supplementary material for: Enzymatic Treatment of Sprouted Wheat and Buckwheat Modulates In Vitro Starch Digestibility and Technological Quality of Wheat Bread
Source: Foods. 2026 Nov 5;15(21):3976. doi: 10.3390/foods15213976 (PMC13763152; doi:10.3390/foods15213976)
Supplement: Supplementary file 1 [file foods-15-03976-s001.zip › foods-4640040-supplementary.pdf]

Table S1. Changes in the carbohydrate composition of wheat and buckwheat during germination (0–48 h)

Cereal species	Germination duration, h	Total starch, %	Reducing sugars (DNS), %	RDS, % of total starch	SDS, % of total starch	RS, % of total starch
Wheat	0	63.1 ± 1.0^{a}	0.82 ± 0.06^{e}	30.6 ± 1.2^{c}	49.6 ± 2.0^{a}	19.8 ± 1.0^{a}
Wheat	12	61.2 ± 1.2^{ab}	1.46 ± 0.09^{d}	34.6 ± 1.4^{bc}	47.4 ± 1.9^{a}	18.0 ± 0.9^{ab}
Wheat	24	58.9 ± 1.3^{bc}	2.37 ± 0.14^{c}	36.8 ± 1.5^{b}	47.0 ± 1.9^{a}	16.1 ± 0.8^{b}
Wheat	36	56.4 ± 1.0^{cd}	3.42 ± 0.17^{b}	44.9 ± 1.8^{a}	42.0 ± 1.6^{b}	13.1 ± 0.7^{c}
Wheat	48	53.8 ± 1.5^{d}	4.18 ± 0.21^{a}	49.1 ± 2.0^{a}	40.0 ± 1.6^{b}	11.2 ± 0.6^{c}
Buckwheat	0	59.8 ± 1.1^{a}	0.54 ± 0.05^{e}	30.9 ± 1.3^{e}	50.0 ± 2.0^{a}	19.1 ± 1.0^{a}
Buckwheat	12	56.9 ± 1.0^{a}	1.31 ± 0.08^{d}	35.9 ± 1.4^{d}	46.6 ± 1.8^{ab}	17.6 ± 0.9^{a}
Buckwheat	24	53.0 ± 1.4^{b}	2.65 ± 0.13^{c}	42.5 ± 1.7^{c}	42.6 ± 1.7^{bc}	15.1 ± 0.7^{b}
Buckwheat	36	49.1 ± 1.2^{c}	4.08 ± 0.19^{b}	48.3 ± 1.9^{ab}	39.3 ± 1.5^{cd}	12.6 ± 0.6^{c}
Buckwheat	48	45.7 ± 1.3^{d}	5.37 ± 0.22^{a}	52.5 ± 2.1^{a}	36.5 ± 1.4^{d}	10.9 ± 0.6^{c}

Note. Data are presented as mean ± SD (n = 3). Different superscript letters within each cereal species and parameter indicate statistically significant differences among germination times according to Tukey's HSD test ($p \leq 0.05$), as reported in the original manuscript. RDS, rapidly digestible starch; SDS, slowly digestible starch; RS, resistant starch.

Table S2. Changes in the carbohydrate composition of wheat and buckwheat germinated for 24 h during enzymatic treatment under different temperature–time conditions

Cereal species	Temperature, °C	Treatment duration, h	Total starch, %	Reducing sugars (DNS), %	RDS, % of total starch	SDS, % of total starch	RS, % of total starch
Wheat	35	12	42.7 ± 1.1^{b}	4.72 ± 0.25^{e}	52.5 ± 2.5^{c}	35.8 ± 2.1^{a}	11.7 ± 1.0^{a}
Wheat	35	24	34.8 ± 1.3^{c}	7.36 ± 0.34^{c}	64.4 ± 3.5^{b}	27.6 ± 2.0^{b}	8.0 ± 0.9^{b}
Wheat	35	36	28.1 ± 1.2^{d}	10.21 ± 0.42^{b}	72.6 ± 4.5^{ab}	21.4 ± 2.0^{cd}	6.0 ± 0.8^{bc}
Wheat	40	6	46.1 ± 1.1^{a}	5.31 ± 0.24^{e}	57.9 ± 2.6^{bc}	32.1 ± 1.9^{ab}	10.0 ± 0.9^{ab}
Wheat	40	12	36.2 ± 1.2^{c}	8.34 ± 0.35^{d}	68.2 ± 3.4^{ab}	24.9 ± 1.9^{bc}	6.9 ± 0.9^{bc}
Wheat	40	18	28.9 ± 1.3^{d}	11.47 ± 0.43^{a}	75.8 ± 4.6^{a}	19.4 ± 1.9^{d}	4.8 ± 0.7^{c}
Buckwheat	35	12	37.9 ± 1.5^{a}	5.54 ± 0.27^{e}	58.6 ± 3.3^{c}	31.4 ± 2.2^{a}	10.0 ± 0.9^{a}
Buckwheat	35	24	28.4 ± 1.2^{b}	8.93 ± 0.38^{c}	69.4 ± 4.1^{abc}	23.9 ± 2.0^{b}	6.7 ± 0.8^{b}
Buckwheat	35	36	20.6 ± 1.0^{c}	12.34 ± 0.47^{b}	80.6 ± 5.2^{a}	17.0 ± 2.1^{c}	2.4 ± 0.5^{c}
Buckwheat	40	6	40.5 ±	6.24 ± 0.29^{e}	62.0 ±	29.6 ±	8.4 ±

			1.0^{a}		2.5^{bc}	1.9^{a}	0.8^{ab}
Buckwheat	40	12	29.6 ± 1.1^{b}	10.18 ± 0.41^{d}	73.6 ± 3.8^{ab}	20.9 ± 1.9^{bc}	5.4 ± 0.7^{b}
Buckwheat	40	18	21.8 ± 1.2^{c}	13.76 ± 0.50^{a}	81.7 ± 5.5^{a}	15.1 ± 1.6^{c}	3.2 ± 0.5^{c}

Note. Data are presented as mean ± SD (n = 3). The superscript letter designations of statistical significance were transferred from the original table without modification and should be verified against the primary statistical analysis results before submission of the manuscript. The conditions selected for bread preparation were 35 °C for 12 h for wheat and 40 °C for 12 h for buckwheat.

Table S3. Results of three-way analysis of variance for the effects of cereal type, processing method, and ingredient inclusion level on the technological quality characteristics of bread

Parameter	Factor/interaction	F	p
Titratable acidity	Cereal type	31.36	<0,001
Titratable acidity	Processing method	16.00	<0,001
Titratable acidity	Ingredient inclusion level	4.96	0,016
Titratable acidity	Cereal type × processing method	5.76	0,025
Titratable acidity	Cereal type × inclusion level	1.12	0,343
Titratable acidity	Processing method × inclusion level	3.04	0,067
Titratable acidity	Cereal type × processing method × inclusion level	6.24	0,007
Porosity	Cereal type	10.13	0,004
Porosity	Processing method	3.13	0,090
Porosity	Ingredient inclusion level	26.37	<0,001
Porosity	Cereal type × processing method	6.13	0,021
Porosity	Cereal type × inclusion level	11.63	<0,001
Porosity	Processing method × inclusion level	1.62	0,218
Porosity	Cereal type × processing method × inclusion level	38.38	<0,001
Loaf volume	Cereal type	24.15	<0,001
Loaf volume	Processing method	34.45	<0,001
Loaf volume	Ingredient inclusion level	2.18	0,134
Loaf volume	Cereal type × processing method	1.19	0,286
Loaf volume	Cereal type × inclusion level	42.61	<0,001
Loaf volume	Processing method × inclusion level	8.05	0,002
Loaf volume	Cereal type × processing method × inclusion level	15.68	<0,001

Note. Three-way analysis of variance (ANOVA, Type II sums of squares) was performed using a balanced dataset comprising 12 formulations (2 cereal types × 2 processing methods × 3 inclusion levels), with three independent baking runs per formulation. The second series of enzyme-treated samples was not included in this model.

Table S4. Results of two-way analysis of variance (ANOVA): effects of experimental series and ingredient inclusion level.

Cereal species	Parameter	Experimental series, p	Ingredient inclusion level, p	Interaction, p
Wheat	RDS	0,638	<0,001	0,904
Wheat	SDS	0,871	<0,001	0,858
Wheat	RS	0,081	0,052	0,191
Wheat	Loaf volume	<0,001	0,021	<0,001
Wheat	Porosity	0,023	<0,001	0,001
Wheat	Titratable acidity	0,002	0,424	0,741
Buckwheat	RDS	0,465	0,013	0,959
Buckwheat	SDS	0,511	0,048	0,976
Buckwheat	RS	0,353	<0,001	0,885
Buckwheat	Loaf volume	<0,001	<0,001	0,002
Buckwheat	Porosity	0,069	<0,001	0,074
Buckwheat	Titratable acidity	0,014	0,010	0,723

Note. Two-way analysis of variance (ANOVA) was performed separately for bread samples containing wheat and buckwheat. The factors were experimental series (S1 and S2) and ingredient inclusion level (10, 15, and 20%), with n = 3 for each factor combination. The p-values for loaf volume, porosity, and titratable acidity were calculated from the results of individual baking runs. For RDS, SDS, and RS, the p-values were reconstructed from the published rounded mean values and standard deviations (n = 3); therefore, minor differences from calculations based on the original unrounded raw data may occur. Statistical significance was set at $p \leq 0.05$.
